# Effect of spironolactone on the progression of coronary calcification in peritoneal dialysis patients: a pilot study

**DOI:** 10.1590/2175-8239-JBN-2019-0009

**Published:** 2019-08-15

**Authors:** Ana Paula Santana Gueiros, José Edevanilson de Barros Gueiros, Karina Tavares Nóbrega, Eveline Barros Calado, Marina Cadena da Matta, Leuridan Cavalcante Torres, Alex Sandro Rolland Souza, Dulce Elena Casarini, Aluizio Barbosa de Carvalho

**Affiliations:** 1 Instituto de Medicina Integral Professor Fernando Figueira Serviço de Nefrologia RecifePE Brasil Instituto de Medicina Integral Professor Fernando Figueira, Serviço de Nefrologia, Recife, PE, Brasil.; 2 Instituto de Medicina Integral Professor Fernando Figueira Serviço de Radiologia RecifePE Brasil Instituto de Medicina Integral Professor Fernando Figueira, Serviço de Radiologia, Recife, PE, Brasil.; 3 Instituto de Medicina Integral Professor Fernando Figueira Departamento de Pesquisa Clínica RecifePE Brasil Instituto de Medicina Integral Professor Fernando Figueira, Departamento de Pesquisa Clínica, Recife, PE, Brasil.; 4 Universidade Federal de São Paulo São PauloSP Brasil Universidade Federal de São Paulo, Disciplina de Nefrologia, São Paulo, SP, Brasil.

**Keywords:** Renal Insufficiency, Chronic, Vascular Calcification, Peritoneal Dialysis, Spironolactone

## Abstract

**Introduction:**

There is evidence that aldosterone plays a role in the pathogenesis of vascular calcification. The aim of this study was to evaluate the effect of spironolactone, a mineralocorticoid receptor antagonist, on the progression of coronary calcification (CC) in peritoneal dialysis patients and to identify the factors involved in this progression.

**Methods:**

Thirty-three patients with a coronary calcium score (CCS) ≥ 30, detected through multi-detector computed tomography (MDCT) and expressed in Agatston units, were randomly assigned to a group receiving 25mg spironolactone per day for 12 months (spironolactone group) and a control group not receiving this drug. The primary outcome was a percentage change in CCS from baseline to end of the study (relative progression), when a further MDCT was conducted. Patients who had progression of CC were compared with those who did not progress.

**Results:**

Sixteen patients, seven in the spironolactone group and nine in the control group, concluded the study. The relative progression of the CCS was similar in both groups, 17.2% and 27.5% in the spironolactone and control groups respectively. Fifty-seven percent of the treated patients and 67% of those in the control group presented progression in the CC scores (*p* = 0.697). Progressor patients differed from non-progressors because they presented higher levels of calcium and low-density lipoprotein cholesterol and lower levels of albumin.

**Conclusion:**

In peritoneal dialysis patients, spironolactone did not attenuate the progression of CC. However, large-scale studies are needed to confirm this observation. Disorders of mineral metabolism and dyslipidemia are involved in the progression of CC.

## INTRODUCTION

Vascular calcification (VC) is prevalent in dialysis patients and is associated with mortality.^1^^,^^2^ The mechanisms involved in chronic kidney disease (CKD) VC are still not fully understood. It is an active process, in which a series of conditions, such as mineral and bone metabolism disorders and inflammation interfere with the vascular microenvironment and interact with promoters and inhibitors of calcification.

Identification of the mineralocorticoid receptor (MR) in cell of the vascular smooth muscle layer (VSMC)^3^^,^^4^ has extended knowledge regarding the action of aldosterone thereby shedding new light on the pathogenesis of VC. Klotho-depleted mice, characterized by an excessive concentration of calcitriol, hypercalcemia and hyperphosphatemia, suffer from hyperaldosteronism.^5^ Patients with CKD also present with heightened levels of aldosterone,^6^ severe klotho deficiency, resistance to the action of fibroblast growth factor 23 (FGF-23) and vascular damage. The involvement of aldosterone in VC was clearly demonstrated when klotho-depleted mice treated with spironolactone showed increased survival rates and decreased VC, without altering the levels of calcitriol, FGF-23, calcium and phosphorus. This study demonstrated that VC mediators, such as Pit-1, a sodium-dependent phosphorus transporter, TNF-α, the Cbfa-1/Runx2 (core-binding factor subunit 1 alpha/runt-related transcription factor 2) protein and alkaline phosphatase were present in increased levels in the aorta of these mice and that transcriptional levels decreased after treatment with spironolactone.^7^ Later authors also reported that inhibition of MR suppressed the osteogenic transformation of aortic VSMC in uremic mice.^8^

Recently, it has also been demonstrated that hyperphosphatemia increases the expression of CYP11B2, an enzyme involved in the synthesis of aldosterone present in human VSMC. A deficiency or inhibition of the action of this enzyme inhibits the osteoinductive pathway and the calcification caused by hyperphosphatemia.^9^ While the quantity of aldosterone produced by VSMC is low and does not affect serum levels, it is nonetheless sufficient to act on the local MR and trigger osteogenic stimuli.^9^

Since VC is indisputably a marker of cardiovascular outcomes, there is a need to investigate procedures that impede its development or attenuate its progression. The aim of the present study was to evaluate the effect of spironolactone on the progression of CC in patients on peritoneal dialysis (PD), and to identify factors involved in this progression.

## PATIENTS AND METHODS

The present study was conducted in the Nephrology Division at IMIP, between November 2014 and November 2016, in accordance with the principles of the Declaration of Helsinki and was approved by the IMIP ethics committee. All patients signed the informed consent forms. This study was registered at ClinicalTrials.gov (NCT03314493).

### SUBJECTS

Patients, aged 18 years or older, on PD for at least 6 months and with a CCS ≥ 30 were eligible for the study. Patients were excluded if they had taken spironolactone up to three months prior to recruitment, presented with hypotension, defined as systolic blood pressure < 100 mmHg and/or diastolic blood pressure < 60 mmHg, had presented in the past three months with mean serum potassium > 6 mEq/L, reported malignant neoplasms or serious liver disease, had undergone heart surgery or stent placement, presented with cardiac arrhythmia or were pregnant.

At the time of inclusion in the study, the majority of the patients (82%) were in automated PD and only 3 patients used dialysate with low calcium concentration (2.5 mEq/L), 1 in the spironolactone group and 2 in the control group.

### STUDY DESIGN

**Figure 1:**
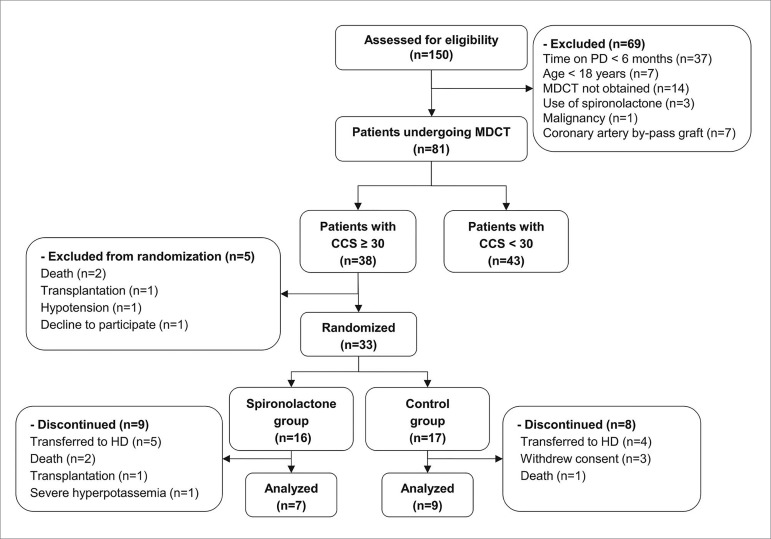
Enrollment, randomization, and follow-up of study patients. PD, peritoneal dialysis; MDCT, multidetector computed tomography; CCS, coronary calcium score; HD, hemodialysis.

This study was a prospective, randomized, open label, controlled, single-center trial. One hundred and fifty patients were assessed, 81 of whom underwent a multi-detector computed tomography (MDCT). Thirty-eight (47%) patients presented with a CCS ≥ 30, while 34 (53%) were not considered calcified, with a CCS < 30. Thirty-three patients were randomly assigned, at a ratio of 1:1, to the spironolactone or the control group (Figure 1). Randomization was conducted through a list generated by “Random Allocation” 1.0, in blocks of 20 and using sealed envelopes.

### INTERVENTION

Patients in the treatment group received a 25 mg oral dose of spironolactone per day for 12 months. Patients in the control group did not receive placebo. During follow-up, all patients were assessed monthly to investigate any clinical occurrences and undergo laboratory tests. They were not subject to further dietary restrictions regarding potassium and continued taking their habitual medication, including anti-hypertensives that interfere with the renin-angiotensin-aldosterone system and medications for mineral and bone metabolism disorders, such as oral phosphate binders (calcium carbonate and sevelamer), calcitriol and calcimimetics.

Hyperkalemia, defined as serum potassium > 6 mEq/L, hypotension and gynecomastia, characterized as enlargement of the breasts, with or without pain, were considered adverse effects of spironolactone and an adjustment of the dose to 12.5 mg per day was therefore indicated. In cases where there was no improvement or severe hyperkalemia (serum potassium > 7 mEq/L), the individual discontinued the study. Other losses were caused by withdrawal of consent, transfer to hemodialysis, kidney transplantation and death.

### LABORATORY TESTS

At baseline and monthly: potassium, total calcium, phosphorus and albumin levels were measured, using an Architect C8000 (Abbott, Abbott Park, Illinois, USA) analyzer. At baseline and quarterly: alkaline phosphatase (Architect C8000); total cholesterol, high density lipoprotein cholesterol, low density lipoprotein cholesterol (LDL), triglycerides, C reactive protein (CRP) and 25 (OH) vitamin D levels were measured, using an Architect I2000 (Abbott, Abbott Park, Illinois, USA) analyzer; and intact parathyroid hormone (iPTH) levels were determined using a chemoluminescence method with a reference range of 12-65 pg/mL. At baseline and at the end: aldosterone (immunoenzyme assay method; normal range 2.5-31.5 ng/dL) and fetuin A (ELISA, cat# DY1184; R&D Systems, Inc. Minneapolis, MN, USA; reference value for healthy volunteers cat# DFTA00 473 ± 95 µ/mL) levels were determined. Calcium was corrected using albumin, according to the following formula: corrected calcium = calcium measured + [(4 - albumin) x 0.8]. Women < 50 years took a pregnancy test (β-HCG; immunochromatographic method) before randomization.

### EVALUATION OF THE CORONARY CALCIUM SCORE

Patients underwent a MDCT (Brilliance TM CT, Philips Medical Systems Nederland B.V.) at baseline and at the end of the observation period. Structures with attenuation coefficients of more than 130 Hounsfield units and a minimum area of 1mm^2^ were considered foci of calcification. The CCS was the sum of all these areas, calculated using the Agatston method. The images were analyzed using a Workstation (Extended Brilliance TM Workspace, Philips Healthcare Nederland B.V.) by an experienced researcher blind to the other study data.

Based on the initial CCS, patients with a score of ≥ 30 were considered to have CC. The value ≥ 30 was used because the inter-exam variability was reported as being equal to or less than 15% with a standard deviation of 10%, and the ability of detecting the progression of calcification was more precise in patients with intermediate to higher scores, since the absolute error in measuring the score was close to the measurements of patients with low scores (1 to 30).^10^ The absolute progression of the CCS was calculated as the difference between the initial and final CCS and the relative progression as the ratio between the absolute progression and the initial CCS multiplied by 100. Progression was considered as existing in patients with a relative progression > 15% and not in those with a relative progression of ≤ 15%.^11^ Investigation of the factors involved in the progression of CCS was carried out by dividing the patients into progression and non-progression groups.

### CLINICAL OUTCOMES

The primary outcome was a percentage change in the CCS from baseline to the end of the study (relative progression). Secondary outcomes included absolute progression, changes in laboratory parameters over the 12 months, frequency of adverse effects of spironolactone, a need to reduce the dose and causes of discontinuing the study.

### SAMPLE SIZE

This study was considered as preliminary, a consecutive convenience sample was obtained for patients with CCS ≥ 30.

### STATISTICAL ANALYSIS

The variables are expressed as mean ± standard deviation or median and interquartile interval. Numeric variables were compared using the Student’s t test or the Mann-Whitney test, depending on the normality of the distribution, evaluated using the Kolmogorov-Smirnov test. The Fischer’s chi-squared test was used to compare qualitative variables.

The characteristics of the patients in the spironolactone and control groups were compared in order to determine selection bias. Thereafter, the relative progression of the CCS in these groups was compared and adjusted for the baseline characteristics that differentiated the groups using a logistic regression model.

A comparison was made between the baseline laboratory parameters and those after the 12-month period, for both the spironolactone and the control groups. The Student’s t test was used for paired samples when the variable met the conditions for normality, and the Wilcoxon test when it did not.

ANOVA for repeated measurements (MANOVA) was used to examine blood pressure and all laboratory parameters, with the exception of fetuin-A and aldosterone, in the spironolactone and control groups, for the 12-month period. This analysis considered the interaction between treatment and time.

A comparative analysis of the baseline and follow-up characteristics was conducted between the progression and non-progression patients in order to determine the factors associated with the progression of CCS.

The software used for statistical analysis was STATA 12.0. All tests adopted a level of significance of 5% (*p*<0.05).

## RESULTS

### PATIENT CHARACTERISTICS AT BASELINE

Of the 33 patients included in this study, 16 concluded the protocol, seven in the spironolactone group and nine in the control group (Figure 1). The baseline characteristics of the patients who completed the study are presented in Table 1. The groups differed in terms of length of time on PD, longer in the control group, and diastolic blood pressure and serum potassium, both of which were higher in the spironolactone group.

**Table 1:** Baseline demographic, clinical and laboratory characteristics of patients

	Spironolactone group (n=7)	Control group (n=9)	*p* Value
**Age (years)**	69.7± 8.9	61.3 ± 8.6	0.077
**Male, n (%)**	3 (42.9%)	5 (55.6%)	0.614
**Time on peritoneal dialysis (months)**	10 (6;24)	53 (33;72)	0.017
**Etiology of CKD (%)**			0.662
Diabetes mellitus	71.4	33.3	
Hypertension	0	22.2	
Chronic glomerulonephritis	0	11.1	
Unknown	14.3	22.2	
Other	14.3	11.1	
**Urine Volume (mL/24h)**	1000 (400;1800)	0 (0;500)	0.053
**Systolic blood pressure (mmHg)**	134.3 ± 18.1	124.4 ± 21.9	0.353
**Diastolic blood pressure (mmHg)**	85.7 ± 7.9	76.7 ± 8.7	0.049
**Coronary calcium score (AU)**	359 (105;490)	422 (161;1125)	0.596
**Comorbidities**			
Smoking	3 (42.9)	4 (44.4)	0.949
Diabetes mellitus	5 (71.4)	3 (33.3)	0.157
Hypertension	7 (100)	8 (88.9)	0.562
Coronary artery disease	2 (28.6)	0 (0)	0.175
Dyslipidemia	4 (57.1)	5 (55.6)	0.671
Peripheral vascular diesase	0 (0)	0 (0)	-
Cerebrovascular accident	0 (0)	0 (0)	-
**Medications, n (%)**			
ACEI/ARB	5 (71.4)	3 (33.3)	0.157
β-Blocker	4 (57.1)	4 (44.4)	0.5
Statin	5 (71.4)	7 (77.8)	0,608
Furosemide	4 (57.1)	4 (44.4)	0.5
Acetylsalicylic acid	3(42.9)	2 (22.2)	0.365
Calcium carbonate	1 (14.3)	2 (22.2)	0.6
Sevelamer	2 (28.6)	5 (55.6)	0.286
Calcitriol	2 (28.6)	2 (22.2)	0.608
Cinacalcet	1 (14.3)	2 (22.2)	0.6
Cholecalciferol	0 (0)	1 (11.1)	0.562
**Laboratory parameters**			
Total calcium (mg/dL)	9.8 ± 0.4	10.1 ± 1.1	0.489
Phosphorus (mg/dL)	4.4 ± 1.1	4.1 ± 1.3	0.52
Alkaline phosphatase (UI/L)	93 (88;128)	75 (72;92)	0.633
Intact parathyroid hormone (pg/mL)	237 (174;316)	250 (143;354)	0.906
25OH vitamin D (ng/mL)	18.4 ± 7.3	19.3 ± 5.7	0.805
Albumin (g/dL)	3.2 ± 0.3	3.6 ± 0.4	0.09
C- reactive protein (mg/dL)	6.6 (4.6;12.3)	2.8 (2;6.3)	0.157
Potassium (mEq/L)	4.7 ± 0.8	3.9 ± 0.5	0.035
Total cholesterol (mg/dL)	186 ± 29.7	200.1± 32.4	0.386
LDL cholesterol (mg/dL)	108 ± 21.6	111.6 ± 27.2	0.813
HDL cholesterol (mg/dL)	35 ± 10.2	37.2 ± 12.4	0.721
Triglyceride (mg/dL)	210 (106;305)	247 (184-295)	0.874
Fetuin-A (µg/mL)	180 (135;235)	191 (174;206)	0.711
Aldosterone (ng/dL)	21.5 (17;27.3)	34.7 (23.5;36.8)	0.223

Values are expressed as number (percentage), median (25th percentile; 75^th^ percentile) and mean ± SD. CKD: chronic kidney disease; ACEI: angiotensin-converting enzyme inhibitor; ARB: angiotensin receptor blocker; LDL: low density lipoprotein; HDL: high density lipoprotein.

Initially, hypoalbuminemia was observed in 50% of patients, high levels of CRP in 46.6%, and high levels of total cholesterol, LDL and triglycerides of 37.5%, 66.6% and 75% respectively. The iPTH levels were lower than 150 pg/mL in 26.6% and higher than 150 pg/mL in 66.6%. Only one patient presented iPTH higher than 600 pg/mL.

### THE INFLUENCE OF SPIRONOLACTONE ON CLINICAL AND LABORATORY PARAMETERS

No differences were observed in the final laboratory parameters between the spironolactone and control groups. In the control group, albumin was lower (*p*=0.007) at the end of the study. Levels of fetuin-A increased in both groups (Table 2).

**Table 2:** Laboratory parameters of the patients at baseline and end of study

	Spironolactone group (n=7)		Control group (n=9)	
	Baseline	Final	Baseline	Final
Total calcium (mg/dL)	9.8 ± 0.4	9.7 ± 1.1	10.1 ± 1.1	9.7 ± 0.7
Phosphorus (mg/dL)	4.4 ± 1.1	4.6 ± 0.9	4.1 ± 1.3	4 ± 1.5
Alakaline phosphatase (UI/L)	93 (88;128)	86 (51;128)	75 (72;92)	87 (61;108)
Intact parathyroid hormone (pg/mL)	237 (174;316)	28 (23;229)	250 (143;354)	179 (154;301)
25OH vitamin D (ng/mL)	18.4 ± 7.3	19.1 ± 7.8	19.3 ± 5.7	19.2 ± 6.1
Albumin (g/dL)	3.2 ± 0.3	3.5 ± 0.4	3.6 ± 0.4^b^	3.4 ± 0.4
C- reactive protein (mg/dL)	6.6 (4.6;12.3)	3.1 (2.2;10.5)	2.8 (2;6.3)	2.4 (1.5;5.4)
Potassium (mEq/L)	4.7 ± 0.8	5 ± 0.9	3.9 ± 0.5	4.1 ± 0.9
Total cholesterol (mg/dL)	186 ± 29.7	211 ± 66.9	200.1 ± 32.4	220.1± 46.6
LDL cholesterol (mg/dL)	108 ± 21.6	111.3 ± 55.6	111.6 ± 29.7	118.9 ± 35.2
HDL cholesterol (mg/dL)	35 ± 10.2	31.8 ± 7.4	37.2 ± 12.4	36.8 ± 14.4
Triglyceride (mg/dL)	210 (106;305)	208 (185;378)	247 (184;295)	206 (183;257)
Fetuin-A (µg/mL)	180 (135;235)^a^	363 (236;414)	191 (174;206)^b^	336 (274;360)
Aldosterone (ng/dL)	21.5 (17;27.3)	29.7 (22;64.6)	34.7 (23.5;36.8)	24 (18.1;29.5)

Values are expressed as median (25^th^ percentile; 75^th^ percentile) and mean ± SD. LDL: low density lipoprotein; HDL: high density lipoprotein.

a Spironolactone group baseline vs. final (*p*<0.05);

b Control group baseline vs. final (*p*<0.05).

MANOVA demonstrated that only albumin behaved differently during follow-up in the two groups, in terms of interaction between treatment and time. There was an increase in the serum levels of patients in the spironolactone group and a decrease in the control group (*p*=0.007).

### PRIMARY AND SECONDARY OUTCOMES

There was no difference in relative progression between the spironolactone and control groups, even when adjusted for the length of time on PD, diastolic blood pressure and potassium. There was also no difference in absolute progression. There was an absolute increase in the CCS in both groups (Table 3). Progression of CCS occurred in 57.1% and 66.7% of patients in the spironolactone and control groups respectively.

**Table 3:** Progression of the coronary calcium score

	Spironolactone group (n=7)	Control group (n=9)	*p* Value^b^	*p* Value^c^
**Coronary calcium score (AU)**				
Baseline	359 (105;490)	422 (161;1125)	0.596	
Final	385 (144;900)	932 (228;1323)	0.427	
***p* Value^a^**	0.042	0.011		
**Absolute progression (AU)**	26.2 (16;253.4)	77.3 (46.2;398.6)	0.27	0.824
**Relative progression (%)**	17.2 (4.2;84)	27.5 (14.1;43)	0.491	0.772

Values are expressed as median (25^th^ percentile; 75^th^ percentile);

a intragroup comparison;

b comparison between groups; c comparison between groups adjusted for time on peritoneal dialysis, diastolic blood pressure and potassium.

c comparison between groups adjusted for time on peritoneal dialysis, diastolic blood pressure and potassium.

There was no difference in the behavior of potassium between the two groups in the interaction between treatment and time. There was also no difference in episodes of hyperkalemia between the groups, whereby there were four episodes amongst treated patients and three in the control group. Only one patient, from the spironolactone group, discontinued the study due to severe hyperkalemia. One patient from each group developed hypotension and there were no episodes of gynecomastia. Two patients in the treatment group needed to reduce the dosage of spironolactone to 12.5 mg per day, one because of hypotension, the other because of hyperkalemia. Both patients however concluded the study.

Seventeen patients (51.5%) discontinued the study, nine in the spironolactone group and eight in the control group. The reasons for discontinuation were similar (Figure 1). Peritonitis non-responsive to clinical treatment was a significant cause of transfer to hemodialysis. The frequency of peritonitis was higher in the spironolactone group (*p*=0.026). Two patients in the spironolactone group died of acute myocardial infarction and one in the control group of complications involving infection.

### BASELINE AND FOLLOW-UP CHARACTERISTICS OF PROGRESSION PATIENTS

The patients in the progression and non-progression groups were differentiated by levels of calcium (*p*=0.001) and LDL (*p*=0.009), both of which were higher in the progression group (Table 4). With regard to the annual mean of laboratory tests (follow-up values), progressor patients presented higher serum calcium levels (*p*=0.004) and lower albumin (*p*=0.006) when compared to non-progressors (Table 4).

**Table 4:** Characteristics of patients by coronary calcium score progression

	Baseline		Follow-up	
	Progression group n=10	Non-progression group n=6	Progression group n=10	Non-progression group n=6
**Age (years)**	66.9 ± 7.2	61.8 ± 12.5		
Male (%)	40	66.7		
**Time on peritoneal dialysis (months)**	30 (16;53)	24 (10;72)		
**Comorbidities (%)**				
Diabetes mellitus	50	50		
Hypertension	100	83.3		
Dyslipidemia	40	83.3		
Smoking	60	50		
**Systolic blood pressure (mmHg)**	133 ± 18.9	121.7 ± 22.3		
**Diastolic blood pressure (mmHg)**	81 ± 8.7	80 ± 10.9		
**Coronary calcium score (AU)**	333 (120;925)	367 (85;1125)	595 (227;1323)	388 (94;1119)
**Laboratory parameters**				
Total calcium (mg/dL)	10.4 ± 0.6^a^	9.2 ± 0.4	9.7 ± 0.5^b^	9 ± 0.3
Phosphorus (mg/dL)	4.4 ± 1	4 ± 1.5	4 ± 0.8	4 ± 1.3
Alakaline phosphatase (IU/L)	91.5 (75;104)	82.5 (72;127)	88 (62;122)	74 (64;87)
Intact parathyroid hormone (pg/mL)	253 (194;421)	143 (137;280)	218 (173;347)	175 (101;273)
25OH vitamin D (ng/mL)	17.6 ± 3.9	21.4 ± 8.8	19.6 ± 7.3	25.8 ± 8.4
Albumin (g/dL)	3.35 ± 0.3	3.58 ± 0.54	3.3 ± 0.3^b^	3.7 ± 0.1
C- reactive protein (mg/dL)	5.4 (2.8;7.6)	2 (1.8;7)	3.5 (2.9-10)	4 (2.2;6.3)
Potassium (mEq/L)	4.16 ± 0.74	4.6 ± 0.83	4.2 ± 0.8	4.5 ± 0.7
Total cholesterol (mg/dL)	197 ± 28.5	188.8 ± 37.2	208 ± 40.9	173 ± 31.2
LDL cholesterol (mg/dL)	119.6 ± 17.6^a^	81.7 ± 18	119.8 ± 27.3	75.1 ± 16.8
HDL cholesterol (mg/dL)	37.1 ± 13.1	34.8 ± 7.1	35.4 ± 13.0	36.4 ± 4.4
Triglyceride (mg/dL)	188 (119;295)	257 (210;447)	200 (176;211)	176 (162;219)
Fetuin-A (µg/mL)	194 (160;229)	178 (174;206)	320 (219;385)	384 (274;372)

Values are expressed as number (percentage), median (25^th^ percentile; 75^th^ percentile) and mean ± SD. Follow-up values are the means for all doses for each parameter during the study. The figures for the coronary calcium score and fetuin-A are for twelve months.

a Progression group vs. non-progression group at baseline (*p*<0,05);

b Progression group vs. non-progression group on follow-up (*p*<0,05).

At baseline, 10% of patients in the progression group had iPTH lower than 150 pg/mL and 80% higher than 150 pg/mL, with only one patient in this group with iPTH higher than 600 pg/mL. In the non-progression group, 40% of patients had iPTH lower than 150 pg/mL, compared to 60% with levels higher than 150 pg/mL and none higher than 600 pg/mL.

## DISCUSSION

This is the first study to prospectively evaluate the effect of the use of spironolactone on the progression of CC in patients undergoing PD. The main result was the failure of spironolactone to attenuate the progression of CCS.

Chronic kidney disease is a state of hyperaldosteronism, as observed in our patients. Studies have indicated the benefit of using MR antagonists for cardiovascular outcomes, both in the general population and in patients with CKD.^12^^,^^13^ Furthermore, it has been demonstrated experimentally that MR blocking is capable of modifying pathways involved in the development of VC.^8^ These observations raise the following question: is the cardiovascular benefit of MR antagonists also related to the development and progression of CC?

The present study has not revealed any benefit from inhibiting the action of aldosterone in the progression of CC. Indeed, in most patients, there was a substantial progression of CC. There are no studies with similar methodologies with which to compare the results. To date, just one clinical study has evaluated the effect of spironolactone on VC in dialysis patients. Nitta et al. demonstrated, after 3 years of observing of 5 patients on hemodialysis, that the use of spironolactone reduced aortic calcification. This study however, did not have a control group.^14^ Another study, aiming to evaluate the effect of spironolactone on the progression of thickening of the tunica intima and media of the carotid, observed that spironolactone reduced progression in 53 patients undergoing hemodialysis.^15^

Similar to other studies,^16^^,^^17^ CC was found to be prevalent in our patients, even when limiting selection criteria were applied. Patients with CKD are prone to cardiovascular disease, since the prevalence of diabetes mellitus, hypertension and dyslipidemia is high in this population. In fact, the main cause of CKD in our patients was diabetes and the majority presented with hypertension and dyslipidemia and were elderly. These comorbidities, advanced age, a longer time on dialysis and mineral and bone metabolism disorders are associated with the development and progression of CC.^17^^-^19

We observed the influence from the use of spironolactone on various clinical and laboratory parameters involved in VC and analyzed the association between these and the progression of CC. Given the two-way relation between aldosterone and the parathyroid hormone, characterized by mutual stimulation of the synthesis of both hormones,^20^^-^22 changes in the levels of iPTH may have occurred as a result of taking spironolactone. However, no alterations were demonstrated in the serum levels of iPTH, calcium, phosphorus, alkaline phosphatase or 25 (OH) vitamin D with the use of spironolactone. Nitta et al. did not observe any changes in serum levels of iPTH or the calcium x phosphorus product with spironolactone.^14^ Vukusich et al. also detected no changes in levels of calcium, phosphorus or iPTH resulting from the use of this drug.^15^ The EPATH study, which examined the effect of eplerenone, a selective MR antagonist on serum levels of iPTH in patients with primary hyperparathyroidism, found no changes in the levels of this hormone.^23^

The present study has demonstrated that mineral metabolism played a significant role in the progression of CC, as has been well established by other studies.^18^^,^^24^ In fact, serum levels of calcium remained higher in the progression patients. Initially, the iPTH levels of the patients were within a range considered adequate for dialysis.^25^ Bone remodeling disorders, both adynamic bone disease and secondary hyperparathyroidism, are strongly associated with VC.^26^^,^^27^ Since no bone biopsy was performed, it was not possible to measure the role of these disorders in the progression of CC.

Inflammation, malnutrition and oxidative stress contribute to the high prevalence of cardiovascular disease in patients on dialysis.^28^ Inflammation, measured using CRP, is associated with the progression of VC in such patients.^29^^,^^30^ Aldosterone has also been implicated as a cause of inflammation and fibrosis, by way of both genomic and non-genomic action.^31^^,^^32^ Tatsumoto et al. demonstrated that spironolactone reduces serum levels of TNF-α, thereby inhibiting inflammatory pathways and helping to retard the progression of VC in uremic rats.^8^ Nitta et al. demonstrated that a reduction in aortic calcification, with a treatment of spironolactone, was associated with reduced levels of inflammatory markers, such as osteopontin.^14^ Although the patients in our study presented inflammation, it was not possible to demonstrate the anti-inflammatory effect of spironolactone, as observed through serum CRP levels at the end of the study. Serum albumin is a marker of nutritional status as is acute-phase inflammation protein. The high levels of CRP and hypoalbuminemia present in our patients may thus reflect the presence of malnutrition-inflammation-atherosclerosis (MIA syndrome), as they were patients with calcification that progressed significantly within a short period of time. These observations are corroborated by the fact that the albumin levels of progression patients were significantly lower than those of the non-progression group throughout the follow-up period. Other authors have reported that patients with calcification, including those on PD, have lower albumin levels than those without calcification.^33^^,^^34^ When the effect of spironolactone on albumin was evaluated, an increase in serum level of the treatment group was demonstrated, which may have reflected a beneficial effect of the MR blocking on inflammatory status.

Fetuin-A is a potent circulating inhibitor of calcification and its serum levels are lower in patients on dialysis, correlating negatively with CCS and becoming a predictor of mortality.^35^^,^^36^ Examining MIA syndrome in patients on DP, Wang et al. reported that, when fetuin-A was stratified into terciles, CRP levels were higher in the lower terciles, demonstrating the role of fetuin-A as an inflammatory marker.^36^ The low serum levels of fetuin-A in our patients further corroborates the presence of MIA syndrome. The use of spironolactone did not bring about any difference in serum levels of fetuin-A between the treatment and control groups.

Dyslipidemia is a classic risk factor for cardiovascular disease and is associated with VC.^17^^,^^37^ Inhibition of MR did not alter serum levels of lipid markers but the influence of dyslipidemia in the progression of CC was confirmed.

There was a significant loss of patients during follow-up and this greatly influenced the results and represented a significant limitation of the present study. The main cause of this was transfer to hemodialysis, which in most cases was due to peritonitis non-responsive to treatment. A higher frequency of peritonitis was found in patients treated with spironolactone, but this would seem to be an incidental result, since there is no justification for such a finding in the literature.^38^^-^40 The most dreaded adverse effect of spironolactone is hyperkalemia. Aldosterone increases renal excretion of potassium and thus patients on dialysis with urinary output may develop hyperkalemia when using MR antagonists. Clinical trials using spironolactone in PD patients, using different definitions of hyperkalemia, were included in a recent systematic review/meta-analysis to evaluate the safety and efficacy of the use of MR antagonists in dialysis, which showed that the use of MR antagonists was significantly associated with hyperkalemia.^41^ The present study observed no significant differences in serum potassium over time between treatment group and control patients. There was also no difference between these patients regarding episodes of hyperkalemia, although one severe episode of hyperkalemia did occur in the spironolactone group. Our study demonstrated no distinct change in blood pressure related to spironolactone. Other studies have produced various results for arterial pressure, although with observations more favorable towards reducing pressure levels.^41^ Gynecomastia, considered an antiandrogenic effect of spironolactone, was not encountered in any of the patients. Despite the small sample size, in general terms, the use of spironolactone was found to be safe. Two patients required a reduction in the dosage to 12.5mg per day and concluded the study. It should be noted that a dose of 12.5mg per day inhibits MR, as shown in other studies.^12^^,^^42^

The present study had significant limitations: the low number of participants, the high number of losses during follow-up, the short observation period and the fact that it was not double-blind. It should therefore be considered a preliminary study, to be continued or followed by others with more appropriate methodologies, with a view to ascertaining whether MR blocking helps to attenuate the progression of CV.

In summary, this study has demonstrated that CC is highly prevalent and has a high progression rate in PD patients. Because of the previously mentioned limitations, it is not possible to draw conclusions about the effect of spironolactone on the progression of CC in this population. Furthermore, we have shown that, even in dialysis patients with residual diuresis, the use of spironolactone is feasible, although serum potassium needs to be controlled. The present study has underlined the need for continued monitoring of VC risk factors such as mineral and bone metabolism disorders, dyslipidemia and inflammation.

## References

[1] Blacher J, Guerin AP, Pannier B, Marchais SJ, London GM. Arterial Calcifications, arterial stiffness, and cardiovascular risk in end-stage renal disease. Hypertension 2001;38:938-42. 10.1161/hy1001.096358 11641313

[2] London GM, Guérin AP, Marchais SJ, Métivier F, Pannier B, Adda H. Arterial media calcification in end-stage renal disease: impact on all-cause and cardiovascular mortality. Nephrol Dial Transplant 2003;18:1731-40. 10.1093/ndt/gfg414 12937218

[3] Williams JS. Evolving research in nongenomic actions of aldosterone. Curr Opin Endocrinol Diebetes Obes 2013;20:198-203. 10.1097/MED.0b013e328360c200 23519092

[4] Wu SY, Yu YR, Cai Y, Jia LX, Wang X, Xiao CS, et al. Endogenous aldosterone is involved in vascular calcification in rat. Exp Biol Med (Maywood) 2012;237:31-7. 10.1258/ebm.2011.011175 22185918

[5] Fischer SS, Kempe DS, Leibrock CB, Rexhepaj R, Siraskar B, Boini KM, et al. Hyperaldosteronism in Klotho-deficient mice. Am J Physiol Renal Physiol 2010;299:F1171-7. 10.1152/ajprenal.00233.2010 PMC3774497 20719979

[6] Hené RJ, Boer P, Koomans HA, Mees EJ. Plasma aldosterone concentration in chronic renal disease. Kidney Int 1982;21:98-101. 10.1038/ki.1982.14 7043053

[7] Voelkl J, Alesutan I, Leibrock CB, Quintanilla-Martinez L, Kuhn V, Feger M, et al. Spironolactone ameliorates PIT1-dependent vascular osteoinduction in klotho-hypomorphic mice. J Clin Invest 2013;123:812-22. 10.1172/JCI64093 PMC3561808 23298834

[8] Tatsumoto N, Yamada S, Tokumoto M, Eriguchi M, Noguchi H, Torisu K, et al. Spironolactone ameliorates arterial medial calcification in uremic rats: the role of mineralocorticoid receptor signaling in vascular calcification. Am J Phisiol Renal Phisol 2015;309:F967-79. 10.1152/ajprenal.00669.2014 26336165

[9] Alesutan I, Voelkl J, Feger M, Kratschmar DV, Castor T, Mia S, et al. Involvement of vascular aldosterone synthase in phosphate-induced osteogenic transformation of vascular smooth muscle cells. Sci Rep 2017;7:2059. 10.1038/s41598-017-01882-2 PMC5435689 28515448

[10] Greenland P, Bonow RO, Brundage BH, Budoff MJ, Eisenberg MJ, Grundy SM, et al.; American College of Cardiology Foundation Clinical Expert Consensus Task Force (ACCF/AHA Writing Committee to Update the 2000 Expert Consensus Document on Electron Beam Computed Tomography); Society of Atherosclerosis Imaging and Prevention; Society of Cardiovascular Computed Tomography. ACCF/AHA 2007 Clinical expert consensus document on coronary artery calcium scoring by computed tomography in global cardiovascular risk assessment and in evaluation of patients with chest pain: A report of the American College of Cardiology Foundation Clinical Expert Consensus Task Force (ACCF/AHA Writing Committee to Update the 2000 Expert Consensus Document on Electron Bean Computed Tomography) developed in collaboration with the Society of Atherosclerosis Imaging and Prevention and the Society of Cardiovascular Computed Tomography. J Am Coll Cardiol 2007;23:378-402. 10.1016/j.jacc.2006.10.001 17239724

[11] Raggi P, Callister TQ, Shaw LJ. Progression of coronary artery calcium and risk of first myocardial infarction in patients receiving cholesterol-lowering therapy. Arterioscler Thromb Vasc Biol 2004;24:1272-7. 10.1161/01.ATV.0000127024.40516.ef 15059806

[12] Pitt B, Zannad F, Remme WJ, Cody R, Castaigne A, Perez A, et al. The effect of spironolactone on morbidity and mortality in patients with severe heart failure. Randomized Aldactone Evaluation Study Investigators. N Engl J Med 1999;341:709-17. 10.1056/NEJM199909023411001 10471456

[13] Matsumoto Y, Mori Y, Kageyama S, Arihara K, Sugiyama T, Ohmura H, et al. Spironolactone reduces cardiovascular and cerebrovascular morbidity and mortality in hemodialysis patients. J Am Coll Cardiol 2014;63:528-36. 10.1016/j.jacc.2013.09.056 24184249

[14] Nitta K, Akiba T, Nihei H. Aldosterone blockade and vascular calcification in hemodialysis patients. Am J Med 2003;115:250. 10.1016/s0002-9343(03)00293-6 12935835

[15] Vukusich A, Kunstmann S, Varela C, Gainza D, Bravo S, Sepulveda D, et al. A randomized, double-blind, placebo-controlled trial of spironolactone on carotid intima-media thickness in nondiabetic hemodialysis patients. Clin J Am Soc Nephrol 2010;5:1380-7. 10.2215/CJN.09421209 PMC2924413 20522535

[16] Stompór T, Pasowicz M, Sulłowicz W, Dembińska-Kieć A, Janda K, Wójcik K, et al. An association between coronary artery calcification score, lipid profile, and selected markers of chronic inflammation in ESRD patients treated with peritoneal dialysis patients. Am J Kidney Dis 2003;41:203-11. 10.1053/ajkd.2003.50005 12500238

[17] Ammirati AL, Dalboni MA, Cendorologo M, Draibe SA, Santos RD, Miname M, et al. The progression and impact of vascular calcification in peritoneal dialysis patients. Perit Dial Int 2007;3:340-6. 17468488

[18] Raggi P, Boulay A, Chasan-Taber S, Amin N, Dillon M, Burke SK, et al. Cardiac calcification in adult hemodialysis patients: a link between end-stage renal disease and cardiovascular disease? J Am Coll Cardiol 2002;39:695-701. 10.1016/s0735-1097(01)01781-8 11849871

[19] Barreto DV, Barreto FC, Carvalho AB, Cuppari L, Cendoroglo M, Draibe SA, et al. Coronary calcification in hemodialysis patients: the contribution of traditional and uremia-related risk factors. Kidney Int 2005;67:1576-82. 10.1111/j.1523-1755.2005.00239.x 15780114

[20] Isales CM, Barrett PQ, Brines M, Bollag W, Rasmussen H. Parathyroid hormone modulates angiotesnsin II-induced aldosterone secretion from the adrenal glomerulosa cell. Endocrinology 1991;129:489-95. 10.1210/endo-129-1-489 1647306

[21] Olgaard K, Lewin E, Bro S, Daugaard H, Egfjord M, Pless V. Enhancement of the stimulatory effect of calcium on aldosterone secretion by parathyroid hormone. Miner Electrol Metabol 1994;20:309-14. 7700220

[22] Rosenberg J, Pines M, Hurwitz S. Response of adrenal cells to parathyroid hormone stimulation. J Endocrinol 1987;112:431-7. 10.1677/joe.0.1120431 3031192

[23] Tomaschitz A, Verheyen N, Meinitizer A, Pieske B, Belyavskiy E, Brussee H, et al. Effect of eplerenone on parathyroid hormone levels in patients with primary hyperparathyroidism: results from EPATH randomized, placebo-controlled trial. J Hypertens 2016;34:1347-56. 10.1097/HJH.0000000000000927 27065001

[24] Goodman WG, Goldin J, Kuizon BD, Yoon C, Gales B, Sider D, et al. Coronary artery calcification in young adults with end-stage renal disease who are undergoing dialysis. N Engl J Med 2000;342:1478-83. 10.1056/NEJM200005183422003 10816185

[25] KDIGO clinical practice guideline for diagnosis, evaluation, prevention, and treatment of Chronic Kidney Disease-Mineral and Bone Disorder (CKD-MBD). Kidney disease: Improving Global Outcomes (KDIGO). Kidney Int 2017;7:S1-S59. 10.1038/ki.2009.188 19644521

[26] Barreto DV, Barreto FC, Carvalho AB, Cuppari L, Draibe SA, Dalboni MA, et al. Association of changes in bone remodeling and coronary calcifications in hemodialysis patients: a prospective study. Am J Kidney Dis 2008;52:1139-50. 10.1053/j.ajkd.2008.06.024 18824289

[27] Neves KR, Graciolli FG, dos Reis LM, Graciolli RG, Neves CL, Magalhães AO, et al. Vascular calcification: contribution of parathyroid hormone in renal failure. Kidney Int 2007;71:1262-70. 10.1038/sj.ki.5002241 17410101

[28] Turkmen K, Kayikcioglu H, Ozbeck O, Solak Y, Kayrak M, Samur C, et al. The relationship between epicardial adipose tissue and malnutrition, inflammation, atherosclerosis/calcification syndrome in ESRD patients. Clin J Am Soc Nephrol 2011;6:1920-5. 10.2215/CJN.00890111 PMC3359546 21757644

[29] Jung HH, Kim SW, Ham H. Inflammation, mineral metabolism and progressive coronary artery calcification in patients on haemodialysis. Nephrol Dial Transplant 2006;21:1915-20. 10.1093/ndt/gfl118 16554319

[30] Yamada K, Fujimoto S, Nishiura R, Komatsu H, Tatsumoto M, Sato Y, et al. Risk factors of the progression of abdominal aortic calcification in patients on chronic haemodialysis. Nephrol Dial Transplant 2007;22:2032-7. 10.1093/ndt/gfm031 17395663

[31] Nagase M. Activation of the aldosterone/mineralocorticoid receptor system in chronic kidney disease and metabolic syndrome. Clin Exp Nephrol 2010;14:303-14. 10.1007/s10157-010-0298-8 20533072

[32] Rocha R, Rudolph AE, Frierdish GE, Nachowiak DA, Kekec BK, Blome EA, et al. Aldosterone induces a vascular inflammatory phenotype in the rate heart. Am J Physiol Heart Circ Physiol 2002;283:H1802-10. 10.1152/ajpheart.01096.2001 12384457

[33] Wang AY, Wang M, Woo J, Lam CW, Li PK, Lui SF, et al. Cardiac valve calcification as an important predictor for all-cause mortality and cardiovascular mortality in long-term peritoneal dialysis patients: a prospective study. J Am Soc Nephrol 2003;14:159-68. 10.1097/01.asn.0000038685.95946.83 12506148

[34] Lee MJ, Shin DH, Kim SJ, Oh HJ, Yoo DE, Ko KI, et al. Progression of aortic arch calcification over 1 year is an independent predictor of mortality in incident peritoneal dialysis patients. PLoS One 2012;7:e48793. 10.1371/journal.pone.0048793 PMC3492238 23144974

[35] Ketteler M, Bongartz P, Westenfeld R, Wildberger JE, Mahnken AH, Böhm R, et al. Association of low fetuin-A (AHSG) concentrations in serum with cardiovascular mortality in patients on dialysis: a cross-sectional study. Lancet 2003;361:827-33. 10.1016/S0140-6736(03)12710-9 12642050

[36] Wang AY, Woo J, Lam CW, Wang M, Chan IH, Gao P, et al. Associations of serum fetuin-A with malnutrition, inflammation, atherosclerosis and valvular calcification syndrome and outcome in peritoneal dialysis patients. Nephrol Dial Transplant 2005;20:1676-85. 10.1093/ndt/gfh891 15899935

[37] Allison MA, Wright CM. A comparison of HDL and LDL cholesterol for prevalent coronary calcification. Int J Cardiol 2004;95:55-60. 10.1016/j.ijcard.2003.04.013 15159039

[38] Lin C, Zhang Q, Zhang H, Lin A. Long-term effects of low dose spironolactone on chronic dialysis patients: a randomized placebo-controlled study. J Clin Hypertens (Greenwich) 2016;18:121-8. 10.1111/jch.12628 PMC8031645 26224543

[39] Ito Y, Mizuno M, Suzuki Y, Tamai H, Hiramatsu T, Ohashi H, et al.; Nagoya Spiro Study Group. Long-term effects of spironolactone in peritoneal dialysis patients. J Am Soc Nephrol 2014;25:1094-102. 10.1681/ASN.2013030273 PMC4005296 24335969

[40] Yongsiri S, Thammakumpee J, Prongnamchai S, Tengpraettanakorn P, Chueansuwan R, Tangjaturonrasme S, et al. Randomized, double-blind, placebo-controlled trial of spironolactone for hypokalemia in continuous ambulatory peritoneal dialysis patients. Ther Apher Dial 2015;19:81-6. 10.1111/1744-9987.12219 25196890

[41] Quach K, Lvtvyn L, Baingent C, Bueti J, Garg AX, Hawley C, et al. The safety and efficacy of mineralocorticoid receptor antagonists in patients who require dialysis: a systematic review and meta-analysis. Am J Kidney Dis 2016;68:591-8. 10.1053/j.ajkd.2016.04.011 27265777

[42] Effectiveness of spironolactone added to an angiotensin-converting enzyme inhibitor and a loop diuretic for severe chronic congestive heart failure (the Randomized Aldactone Evaluation Study [RALES]). Am J Cardiol 1996;78:902-7. 10.1016/s0002-9149(96)00465-1 8888663

